# Nanomaterials: A Review about Halloysite Nanotubes, Properties, and Application in the Biological Field

**DOI:** 10.3390/ijms231911518

**Published:** 2022-09-29

**Authors:** Giuseppa Biddeci, Gaetano Spinelli, Paolo Colomba, Francesco Di Blasi

**Affiliations:** Institute for Innovation and Biomedical Research (IRIB), 90146 Palermo, CNR, Italy

**Keywords:** halloysite nanotubes, biocompatibility, drug delivery system, biomedical application

## Abstract

The use of synthetic materials and the attention towards environmental hazards and toxicity impose the development of green composites with natural origins. Clay is one of the candidates for this approach. Halloysite is a natural clay mineral, a member of the Kaolin group, with characteristic tubular morphology, usually named halloysite nanotubes (HNTs). The different surface chemistry of halloysite allows the selective modification of both the external surface and the inner lumen by supramolecular or covalent interactions. An interesting aspect of HNTs is related to the possibility of introducing different species that can be released more slowly compared to the pristine compound. Due to their unique hollow morphology and large cavity, HNTs can be employed as an optimal natural nanocarrier. This review discusses the structure, properties, and application of HNTs in the biological field, highlighting their high biocompatibility, and analyse the opportunity to use new HNT hybrids as drug carriers and delivery systems.

## 1. Introduction

As emerging materials, nanomaterials have attracted much attention over the years due to their small size, but also due to the countless properties that distinguish them. In fact, the nanometric dimensions of material make it assume particular and different chemical-physical properties compared to conventional materials. These different properties, determined by the chemical composition, structure, surface, and increase in surface reactivity in relation to volume, solubility, and state of aggregation, have raised questions about potential human health and environmental risks [1]. In the past few years, there has been a growing interest in research aimed at the development of new organic or inorganic nanocomposites [2,3,4,5]. The attention of the scientific community has been drawn by nano clays, thanks to their natural origin, worldwide abundance, availability, biocompatibility, and sustainability [6,7,8]. Halloysite, largely known as halloysite nanotubes or HNTs, is a natural mineral clay composed of alternating layers of silica and alumina geologically laminated in mesoporous tubular particles with significant adsorption and loading capabilities [9]. Compared to other tubular nanomaterials, HNTs show some advantages in terms of processability and hydrodynamic properties [10]. In fact, for many years, much attention was focused on carbon nanotubes (CNTs) [11,12,13,14], showing that these nanotubes have a high cost, lower water dispersibility, and higher toxicity than HNTs [15]. The physicochemical properties of HNTs were fully described, disclosing their potential for various applications such as biomedicine and catalysis [16,17,18,19]. HNT-based composites are gaining interest in research aimed at the development of biomaterials for drug delivery vehicles in nanomedicine [20]. In this review, we aim to provide an overview of the properties and biomedical applications of halloysite nanotubes as drug delivery systems. We will discuss their possible application in biotechnology through a focus on HNT composites for biomedical applications. Herein, we aim to provide an overview of the structure, properties, and applicative aspects of halloysite nanotubes in the biomedical field. We start with a brief background about HNTs then provide a brief discussion of their biocompatibility and application.

## 2. Halloysite Nanotubes

**Figure 1:**
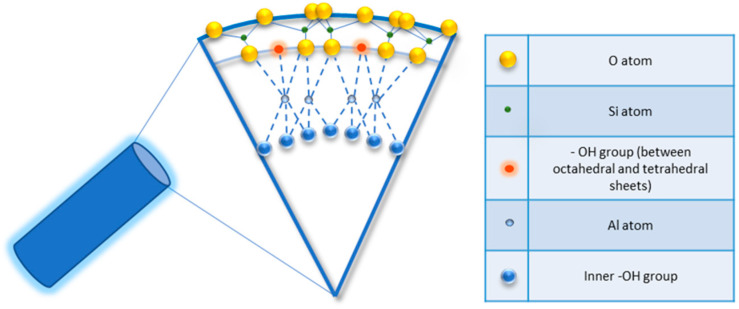
Detail of schematic illustration of the crystalline structure of halloysite nanotubes.

Halloysite is a two-layered aluminosilicate with a chemical composition similar to kaolinite (chemical formula: Al_2_Si_2_O_5_ (OH)_4_·nH_2_O) [21,22], and a hollow tubular structure in the sub-micrometre range [23,24,25,26]. Halloysite was first described in 1826 by the French chemist Pierre Berthier and was later given its name in honour of the Belgian geologist Omalius d’Halloy, who found it in the deposits of Angleur, Belgium [27]. Natural tubular halloysite clay has attracted great interest in materials development because it is one of the few inexpensive nanomaterials available in thousands of tons at a low price [28,29]. The nucleation of halloysite crystals occurs in different parts of the world, because of rock erosion due to weathering, pedogenesis, and hydrothermal alteration of ultramafic rocks [30,31]. New Zealand, Belgium, Brazil, France, China, Australia, and Turkey are rich in deposits of halloysite, and it has been shown that with the variation of the deposits it is possible to observe different characteristics that are maintained within the same deposit. Halloysite is defined as a 1:1 phyllosilicate in which a planar layer of tetrahedral silicates alternates with an octahedral geometry layer; these layers are bound together by oxygen bridges [32]. The fundamental unit for the octahedral sheet consists of three octahedrons. In particular, the siloxane groups are bonded via only one oxygen atom to octahedral rings at the outer part and the apical oxygen of tetrahedra becomes the vertices of octahedra [33]. However, under certain geological conditions, halloysite can also take on forms other than classical tubular. It is also possible to distinguish a spheroidal morphology, flat and almost rolled [34,35]. In the “Dragon Mine” deposits of Utah (USA), halloysite is characterized by a good degree of purity and looks like a white stone that can easily be transformed into soft, fine powder. In some deposits, the presence of metals as contaminants induces a colour change that becomes yellowish or brown [30]. Transmission electron microscopy (TEM) structural analysis has shown that halloysite with predominantly tubular morphology and heterogeneous dimensions is present in New Zealand, the United States, and Australian deposits [36]. A very interesting aspect is linked to the different chemical composition between the inner and outer surfaces, in which there are, respectively, aluminolic groups (Al-OH) that give a positive charge and siloxanes (Si-O-Si) that give a negative charge [37,38]. The charges that characterize the internal and external nanotube surfaces are due to the different dielectric and ionization properties of silicon oxides and aluminium. For pH values of 3–10, the positive charges are distributed in the inner lumen and the negative charges on the external surface; present on the edges is a negative/positive charge. In particular, the tubule lumen is positively charged with pH ≤ 8.5, and the outer surface is negatively charged with pH ≥ 1.5 [39]. Generally, because of the O and OH atoms that carry negative charges, the halloysite nanotube is negatively charged. As a result of the tubular shape, on the outer surface only a few hydroxyl groups are present; these are more concentrated in the internal lumen and therefore are more reactive. In fact, for halloysite, it is possible to classify three types of Al-OH, according to their positioning on the surface, at the ends, and between the octahedral and tetrahedral sheets, as shown in Figure 1. All can be reactive and dissociate according to the pH of the solutions, except those placed between the octahedral and tetrahedral sheets, due to steric hindrance [40].

**Figure 2:**
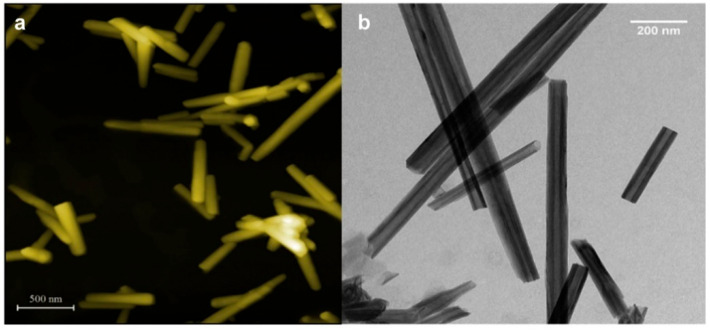
(**a**) Atomic force microscopy; (**b**) TEM images of halloysite nanotubes precipitated from aqueous dispersion. Adapted from [43].

The halloysite nanotubes’ size may vary depending on the extraction site and the purification process they undergo, but they usually have an internal diameter of 10–30 nm, an external diameter of 40–70 nm [41], and a length between 200 and 2000 nm [42] (Figure 2a,b).

Nanotubes with a length between 3 and 5 μm have been found in some deposits, although those with smaller sizes are more interesting from a biological point of view, as they are more suitable for use as drug carriers [44]. Halloysite is chemically like kaolinite, but the halloysite double layers are separated by a monolayer of water molecules. In the hydrated form of halloysite *n* = 2 in the formula Al_2_(OH)_4_Si_2_O_5_·*n*H_2_O. One layer of water molecules is present between the multilayers and is named “halloysite-(10 Å)”, where “10 Å” indicates the d_001_-value of the layers. When the *n* value is 0 (*n* = 0), the halloysite is named dehydrated or “halloysite-(7 Å)” and may be obtained through the loss of the interlayer water molecules [30,45]. The water present in halloysite-(10 Å) can be classified as “hole water” and “associated water”. In the first case, the water molecules are placed on the surface of the tetrahedral sheet with different orientations and make hydrogen bonds with the basal oxygens. The “associated” water has a greater degree of mobility at room temperature and is located at a different level in the interlayer space, with an ice-like configuration and forming hydrogen bonds with each other and/or with inner-surface hydroxyls [46,47,48,49,50]. Generally, HNT surfaces could be classified into three types. The inner lumen surface has a positive charge and is covered by Al-OH groups. It can undergo a variety of covalent modifications by which certain functional groups can be added. This method allows immobilizing several organic groups on the surface of the lumen stably. The external siloxane surface has a negative charge and can be used to establish covalent bonding with molecules such as organosilanes [51]. Moreover, it can be modified by coating with cationic substances, such as polymers, biopolymers, and surfactants. This type of modification can help to improve the dispersibility and biocompatibility of halloysite. Interstate surfaces, held together by hydrogen bridges, can be modified by direct or indirect intercalation of small organic molecules and some monovalent cationic salts. This, therefore, can lead to a weakening of the hydrogen bonds interstate and an increase in the surface between the various layers that can be understood as additional space for loading or adsorption [52].

## 3. Effects of Nanomaterial on Human Health

**Figure 3:**
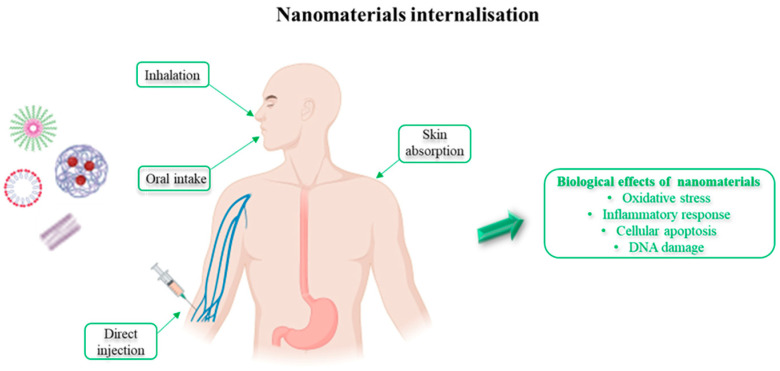
Nanomaterial internalisation and biological effects on the human body.

Despite nanomaterials having potential benefits, their interaction with biological systems may cause unpredicted risk to human life. One concern is the variation in dimensions of nanoparticles, which may affect chemical, thermodynamic, optical, biological, spectroscopic, electronic, and electromagnetic processes, resulting in unexpected modifications [53,54,55]. An increasing exposure to different types of nanomaterials makes it essential to determine their possible negative impact on human health and potential toxic effects. For this reason, it is important to evaluate and characterize the chemical-physical properties, dimensions, solubility, chemical composition, surface area, surface energy, etc. [56]. Among these properties, size and surface are the most important for interaction with biological systems as they determine how the materials will respond [57]. It was observed that the mechanisms for cell internalization depend mainly on the material size [58] and that toxicity may also depend on this aspect. The size of nanomaterials depends on the ability to enter the biological systems, to interact with cellular functions by modifying the structure of macromolecules, and thus interfere with biological functions [59,60]. Nanomaterial internalisation can take place by many paths such as inhalation, oral intake, direct injection, or skin absorption (Figure 3), and is followed by their transportation into organs and their succeeding biological effects, including oxidative stress, inflammatory responses, cellular apoptosis, and DNA damage [61,62].

Nanostructures, once ingested, administered topically, or inhaled, can be transported by blood and accumulated in various organs [63]. Subsequent to entry into the systemic circulation, the absorption of nanoparticles by blood capillaries allows the distribution in various body districts. Depending on their surface characteristics, they can be recognized and degraded by macrophages [64]. Moreover, nanocarrier size also affects the in vivo fate. In fact, particles larger than 200 nanometres are shown to accumulate in the liver and spleen. Instead, nanoparticles smaller than five nanometres are filtered at the renal level [65]. It has been observed that the optimal size of a nano system for application in the biomedical field is around 100 nanometres [66]. In vivo studies have shown that once nanomaterials enter the bloodstream they can reach the central nervous system [67], induce pulmonary inflammatory reactions [68,69], and cause cardiovascular problems [70]. The toxic effects induced in the cells by contact with nanomaterials can be chemical or physical. Chemical mechanisms may include the production of reactive oxygen species (ROS) [71], disruption of electron and ion transport across the cell membrane [72], and lipid peroxidation with a consequent decrease in the fluidity of the membranes [73]. Concerning physical mechanisms, which depend on the size of the nanomaterial and the surface properties [74], these include rupture of the plasma membrane and interruption of related activities such as transport processes [75,76] and misfolding of proteins resulting in loss of their function [77]. The life cycle of nanomaterials in the human body, their metabolism, and their fate in human organs are dependent on the physical and chemical characteristics of the nanomaterials and on their exposure route [78]. The exposure of humans to nanomaterials requires an improved understanding of their potential toxicity to predict the consequences. Future research efforts should be towards developing easy, cost-effective, and highly biocompatible nanomaterials for their application in the biomedical field.

## 4. Halloysite Biocompatibility

Thanks to their rheological properties, high interaction, and high binding capacity with biopolymers, the role of mineral clays as a drug carrier has become the subject of extensive research. The physical-chemical properties, including size, morphology, and surface charge density, which depend on the type of mineral clay and the crystalline structure, are the features that make them interesting [79]. The increased use of halloysite nanotubes, their purification, modification, and large-scale preparation lead to an increasing exposure to human society and environments. Therefore, it is important to do a systematic analysis before their use in biomedical applications and to consider the possible consequential effects of their use on human health. The different interactions of HNTs with living cells, encompassing electrostatic, van der Waals, and ion exchange, as well as cellular response, are decisive in determining the behaviour of halloysite nanotubes in biological systems [80]. Due to their abundance and perceived biocompatibility, in recent years halloysite nanotube toxicity has been evaluated in different in vitro and in vivo models. For example, Sawicka et al. investigated both short- (24 or 72 h) and long-term (seven days) cytotoxic effects of HNTs at doses ranging from 10 to 200 μg/mL on human alveolar carcinoma epithelial cells (A549) and human bronchial epithelial cells (BEAS-2B). After 24 h of exposure, the IC_50_ of HNTs in A549 and BEAS-2B cells was 152 ± 6.4 μg/mL and > 400 μg/mL, respectively. After 72 h of exposure, the IC_50_ values decreased to 49 ± 3 μg/mL in A549 and 45.1 ± 8 μg/mL, in BEAS-2B cells. Thus, the results showed that cytotoxicity of HNTs depends on cell model, dose, and time of exposure [81]. Table 1 shows some of the toxicity threshold values reported for different concentrations of HNTs regarding cell types.

**Figure 4:**
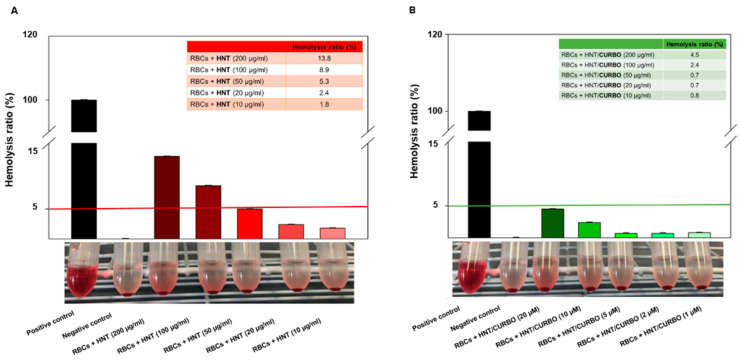
In vitro hemocompatibility assay of (**A**) raw HNT and (**B**) HNT/CURBO after incubation at 37 °C for 12 h. The positive control was in ultrapure water (100% lysis), and negative control was in PBS 1× (0% lysis). International Journal of NanoMedicine 2021:16 4755–4768 Originally published by and used with permission from Dove Medical Press Ltd [88].

Kamalieva et al., evaluated the internalization of pristine halloysite nanotubes using the A549 cell line. To assess the cytotoxicity, the cells were treated for 24 h with increasing concentrations of pristine HNT (ranging from 33 to 900 μg per 10^5^ cells). Once the treatment was finished, the IC_50_ of halloysite nanotubes for A549 cells was determined to be 300 μg per 10^5^ cells by MTT assay [87]. Regarding the nanomaterials for applications in the biomedical field, it is also important to verify their haemolytic potency when contacting the blood. To this end, the hemocompatibility of the hybrid system HNT/CURBO was investigated by exposing human red blood cells (HRBCs) both to pristine halloysite and HNT/CURBO for 24 h at 37 °C. After exposure to different concentrations, a non-noticeable haemolytic effect was observed. The haemolysis percentage values induced by the exposition of HRBCs to different concentrations of HNT/CURBO (ranging from 10 µg/mL to 200 µg/mL) were all less than 5% (Figure 4).

**Figure 5:**
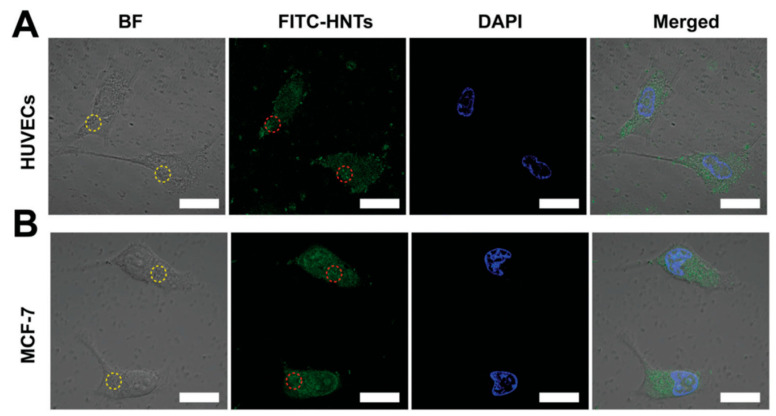
CLSM images of (**A**) HUVECs and (**B**) MCF-7 cells. Cells incubated with FITC-HNTs (50 mg mL^−1^); FITC-HNTs and cell nucleus are indicated in green and blue, respectively. The circles in the images represent the FITC-HNT aggregates. Scale bar = 20 mm. Adapted from [89].

This result suggests that HNTs do not exhibit a significant haemolytic effect [88]. It has also been seen that HUVECs (human umbilical vein endothelial cells) and MCF-7 (human breast cancer) cells show high cell viability after being treated with different concentrations of HNTs for 24 h. For both cell lines, vitality remains above 85% even when the concentration of HNTs reaches up to 200 mg/mL. When incubation times increase (48 h and 72 h), a slight decrease is observed in cell viability. In particular, at 72 h in HUVEC cells viability is 62.1% at the maximum HNT concentration of 200 mg/mL. Furthermore, to investigate the cellular uptake of HNTs in HUVECs and MCF-7, the cells were incubated with FITC-HNTs and monitored using confocal laser scanning microscope (CLSM) as shown in Figure 5 [89].

**Figure 6:**
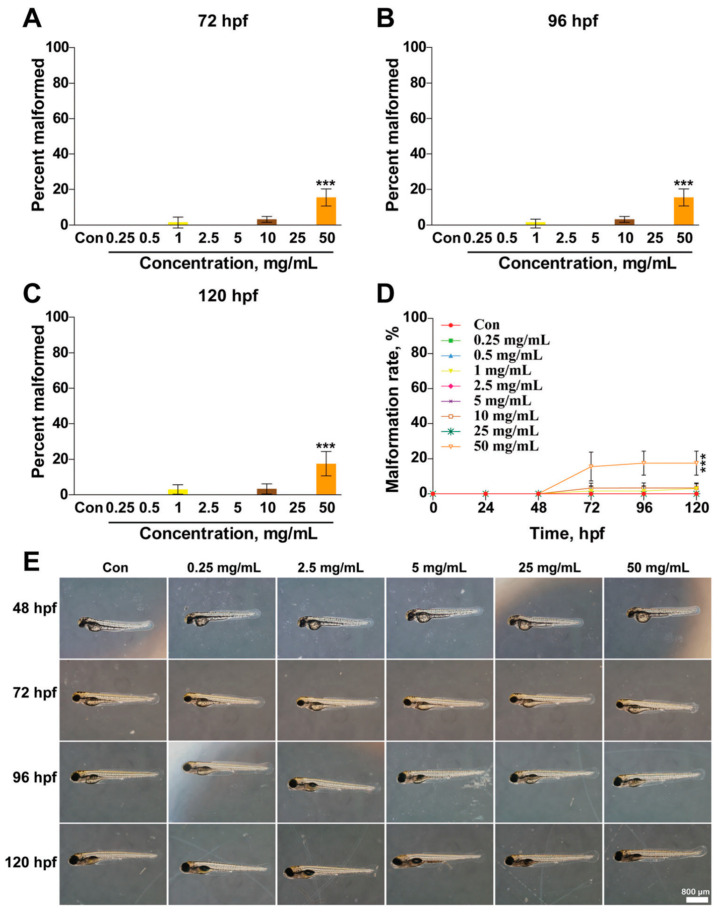
Effects of HNTs on the morphology of developing zebrafish. Embryos were treated with different concentrations of HNTs (0, 0.25, 2.5, 5, 25, and 50 mg mL^−1^) starting from 6 hpf. (**A**–**D**) Percent of malformed zebrafish was analysed at 72, 96, and 120 hpf. (**E**) Morphology of zebrafish larvae treated with HNTs was photographed using a microscope at 48, 72, 96, and 120 hpf. The values are represented as mean ± SD (*n* = 24). Each bar represents the collated data of three separate experiments. The data were analysed using Graph Prim 6 for one-way ANOVA and a Tukey’s post hoc test. *** *p* < 0.001 versus control group. Adapted from [89].

Halloysite nanotube toxicity was further evaluated against human peripheral lymphocytes by means of mitotic index assay. Different concentrations of HNTs (10, 100, 500, and 1000 µg/mL) were incubated with the peripheral lymphocyte culture. The mitotic index assay revealed inhibition of the proliferation of lymphocytes only at the highest concentration (1000 µg/mL) [90]. Despite many in vitro studies indicating that HNTs exhibit a high level of biocompatibility, the in vivo toxicity of HNTs remains unclear. HNT toxicity has been evaluated in in vivo models and in all cases, results show that this nanomaterial is non-toxic or scarcely toxic depending on the tested condition. Several concentrations of HNTs (ranging from 0.25 to 50 mg/mL) were tested towards zebrafish embryos and larvae from 24 h post-fertilization (hpf) to 120 hpf and the results showed that the percent survival of zebrafish embryos and larvae have no significant changes at different developmental stages (24, 48, 72, 96, and 120 hpf) except at the highest concentrations (25 and 50 mg/mL) (Figure 6).

**Figure 7:**
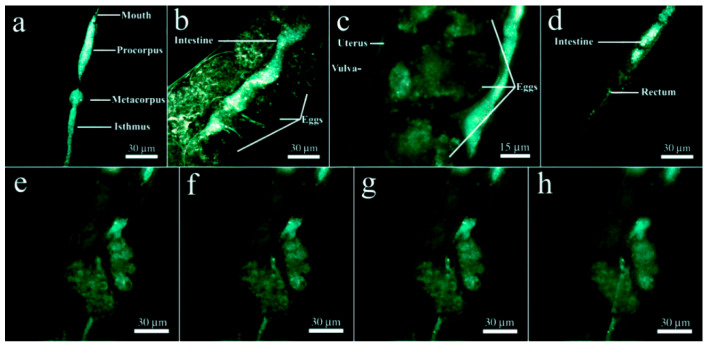
HNT localization in the nematode’s intestines by EDF microscopy: (**a**) inside the foregut; (**b**,**c**) in the midgut; (**d**) inside the hindgut; (**e**–**h**) EDF images of the intestine near the uterus taken at different focal planes demonstrating the localisation of HNTs exclusively inside the intestine. Adapted from [91].

Following the treatment at relatively low HNT concentrations, the percent survival for all the hpf data sets increases slightly above 100%, then the HNTs increase the survival of the zebrafish at low concentrations and have little effect on the survival, proving to be weakly toxic [89]. Fakhrullin et al. evaluated the in vivo toxicity of HNTs by using as a model organism the nematode *Caenorhabditis elegans*. The results showed that HNTs within the investigated concentrations, ranging from 0.05 mg/mL to 1 mg/mL, were not toxic to the nematode. They only induce mechanical stress in the digestive system, which is restored once the treatment is finished. In extended depth of field (EDF) microscopy images, halloysite nanotubes were found exclusively in the alimentary system of the worms (Figure 7) [91].

In addition, a phytotoxic study carried out on *Raphanus sativus* has shown that halloysite nanotubes do not affect the germination process, xylem differentiation, and development, thus demonstrating a high biocompatibility also with respect to plant species [92]. Another study evaluates the biodistribution and pulmonary toxicity of the purified HNTs in mice, following an intragastric administration for 30 days. The results showed that HNT oral administration caused considerable aluminium accumulation mainly in the lungs. Oral administration of HNTs stimulated the growth of the mice at low dose (5 mg/kg BW) with no pulmonary toxicity. When concentrations increased ten times (50 mg/kg BW), the mouse growth was inhibited and resulted in lung inflammation and oxidative stress [93]. An additional study instead showed normal mice weight gain at an oral dose of 5 mg kg^−1^ and a reversible inflammation of the small intestine [94]. It is possible to state that HNTs have been shown to be almost non toxic under the tested conditions, and no cell toxicity makes HNTs suitable as safe materials for biomedical applications.

## 5. Halloysite Nanotube Application in Drug Delivery

Clay minerals are commonly used in the pharmaceutical industry either as excipients or activated ingredients. Indeed, it is known that when drugs and clay mineral are administered simultaneously the absorption capacity reduces by interacting with the drug. These kinds of interactions can represent advantages, for example in drug release [95]. Research has recently focused on the development of new drug delivery systems based on the use of nanomaterials, with the aim of improving the efficacy from the therapeutic point of view and reducing the side effects, especially, in cancer treatment. In several studies HNTs have been used as nanocontainers or nanocarriers for drug delivery. Since unmodified halloysite nanotubes have been shown to establish weak interaction with the drugs, several methods of modification have been developed, for example, tubular entrapment, adsorption, or intercalation [96,97,98]. Commonly, the methods used for the HNT loading consists of three steps. The first step is mixing the clay dry powder with the saturated solution of the guest molecule; the second step is the sonication and stirring of the HNT/guest molecule dispersion; and the last step is the vacuum pumping in/out operation, in which the dispersion is transferred from atmospheric pressure to a vacuum jar. The third step was introduced to optimize the quantity of active molecules loaded inside the nanotubes by keeping the system under vacuum and then cycling it back to atmospheric pressure [99]. Thanks to hydroxyl groups present both on the outer and inner surface, HNTs can be functionalized and used as delivery vehicles of drugs [18,100]. In fact, thanks to the structural properties, high surface area (up to 184.9 m^2^/g), and the large pore volume (up to 0.353 cm^3^/g), over the last few years, halloysite has attracted increasing attention in several areas such as carriers for drug delivery, adsorbents, photocatalysts, etc. [101]. The functional groups of external (Si-OH) and internal (Al-OH) surfaces affect the loading of molecules of interest into HNTs. The negative charge of HNTs at pH above three, is due to silanol group deprotonation. The anionic nature of the external HNT surface enables it to interact with cationic compounds, while aluminol groups located on the internal lumen surface carry a positive charge and support the loading of anionic molecules. Moreover, interactions between HNTs and molecules can take place by van der Waals, hydrogen binding, or other specific interactions [102]. Due to the unique properties mentioned in the previous sections, HNTs have attracted research interest for application in drug delivery. In fact, drug molecules could be encapsulated inside the tube lumen or might be adsorbed on the outer surface [103,104,105]. Furthermore, the tubular morphology allows an increase in tensile and bending strength [106]. Taking advantage of the hollow tubular shape and the large cavity volume, HNTs can be used as desirable natural nanocarriers for biologically active agents. Drug therapy is the main approach in cancer treatment. Due to the poor loading capacity of hydrophobic molecules commonly used in cancer therapy, as for example curcumin, doxorubicin, and paclitaxel, halloysite nanotubes are not considered as an efficient carrier system for cancer drug therapy [107,108,109]. Since the surface of halloysite nanotubes is negatively charged, polycations such as chitosan can be coated onto HNTs. Thanks to this type of approach, drugs released from the HNT lumen may be sustained over a long period of time. Following this reasoning, curcumin was entrapped into the lumen of halloysite with the aid of vacuum suction and release obtaining drug-loaded halloysite nanotubes (DLHNTs). Then, the DLHNTs were coated with chitosan (DLHNTs-CH). The viability assay performed on MCF7 cells showed that polycationic coated HNTs have the potential to serve as a drug carrier [110]. Following surface modification of distearoyl phosphoethanolamine (DSPE), paclitaxel (PTX) is successfully loaded onto HNT surfaces with different inner lumen diameters giving rise to the system DSPE-HNTs-PTX, designed to deliver this drug to cancer cells. The antitumoral effects of the DSPE-HNTs-PTX system were evaluated on MDA-MB-231-bearing mice. The results showed that the system can inhibits tumour growth, suggesting a good anticancer effect [111]. Another study was conducted to evaluate the anticancer effect of chitosan-modified HNT loaded with curcumin-gold hybrid nanoparticles (HNT@CUR-Au/CS). This HNT hybrid system consisted of AuNP which have near-infrared (NIR) responsive property and pH-responsive curcumin release. The anticancer efficacy of HNT@CUR-Au/CS was tested on MCF-7 breast cancer cells showing more effective anticancer activity at pH 5.5 (intracellular tumour environment) than at a pH value of 7.4 (extracellular conditions) [112]. Taheri-Ledari et al. exploiting the properties of HNTs and gold nanoparticles (AuNPs), proposed a new system for controlled release of docetaxel (DTX), a cytotoxic anticancer agent. The DTX@HNT/Au-SORT system is composed of HNTs conjugated with monoclonal antibody as a biologically active agent for targeted drug delivery and small plasmonically active AuNPs included in the HNT pores. In vitro cytotoxicity assay performed on 3T3 (human normal fibroblast) and caov-4 (human ovarian cancer) cell lines showed high selectivity of DTX@HNT/Au-SORT in cell adhesion and internalization. At a concentration of DTX@HNT/Au-SORT equal to 50 μg mL^−1^ the cytotoxicity was approximately 90% for caov-4 cells and 16% for the 3T3 cell line demonstrating that it could be a promising system to use in the treatment of ovarian cancer [113]. In recent years, various methods have been developed to functionalize HNTs and allow them to be used as delivery systems for anticancer drugs. In Table 2 we summarize literature reports with respect to some anticancer drugs entrapped in HNTs.

Changes in the HNTs surface are important to improve their hydrophilicity and compatibility. One of the advantages of the use of clay nanomaterials is the ability to protect drugs against degradation by chemicals and enzymes while extending the drug release rate [130]. In the inner cavity of HNTs is possible to load not only small drug molecules but also proteins, DNA, or antibacterial agents [131,132,133,134]. Various modifications of the HNTs’ lumen can increase the affinity of the drug towards the HNTs, thereby controlling the release rate [135]. Molecules and drugs released from HNTs’ lumen can take place by diffusion from the lumen or by desorption from the external surface. Release from the lumen can be controlled by the tube diameter or by addition of tube-end stoppers [136,137]. The functionalization of HNTs with stimuli-responsive materials, for example with thermosensitive polymers, allows the release profile to be adjusted [138]. Kartogenin is a small molecule that promotes the selective differentiation of multipotent mesenchymal stem cells into chondrocytes, stimulating the repair of damaged cartilage. Unfortunately, like most organic molecules with biological properties, it possesses short-term stability in an aqueous medium. As an efficient treatment for osteoarthritis, halloysite nanotubes have been proposed as a carrier system for potential intra-articular delivery of KGN by means of laponite hydrogel (HNT/KGN/Lap). The cytotoxicity of the hybrid hydrogels was evaluated in human liver HepG2 cells. The efficacy of HNT/Lap hydrogel as a carrier for KGN was proved by in vitro release experiments performed at pH 7.4 and in ex vivo synovial fluid at 37 °C and it was observed that KGN has a slower release in synovial fluid than that of phosphate buffer at pH 7.4 [139]. Alternative approaches can be employed for HNT surface modifications before the loading of drugs. One of these is the modification through (3-Aminopropyl) triethoxysilane (APTES), known for the functionalization of surfaces due to their ease of use and low toxicity. Its role is to introduce a silanol group on the surfaces of halloysite nanotubes to establish a bond with hydroxyl groups [95]. Pristine HNTs and APTES-modified HNTs were tested as drug carriers for loading and release of ibuprofen (IBU) that was encapsulated into the lumen and partially loaded onto the external surface. When unmodified halloysite nanotubes are used as a carrier for ibuprofen, a low loading rate and rapid release is achieved because the only established interactions between HNTs and ibuprofen are weak bonds (Van der Waals). HNT surface modification with APTES increases the loading of IBU by creating an electrostatic attraction between the introduced aminopropyl groups of the grafted APTES and the carboxyl group of ibuprofen and induces a delay in the release from the lumen of nanotubes [140]. In addition to exploiting the HNT lumen for drug encapsulation, it is possible to adsorb or add by covalent bonding molecules on the outer surface of nanotubes. By combining both, it is possible to obtain a system that can release the drug from the external surface initially and subsequently, in a slow and controlled way, that of the lumen. This type of system can be obtained through the functionalization of the external surface, for example, with some linkers. It has been observed that the functionalisation of the outer surface of nanotubes by the addition of triazolium salts is useful for the transport of curcumin. This system, in addition to demonstrating high efficiency for curcumin encapsulation and for controlled and prolonged release capacity, shows cytotoxic effects against different tumour cell lines [141]. The bioavailability of ciprofloxacin (CIP), due to its complexation with iron present in the body, decreases subsequent to administration. Entrapping the drug in a carrier could be a solution. To this end, CIP was loaded onto APTES modified halloysite nanotubes. The result was a high adsorption capacity in modified HNTs for CIP (70% ± 1.7%), compared to that of pristine halloysite nanotubes [142]. A multi-layered polylactic acid (PLA)/HNT porous membrane encapsulated with gentamicin was prepared for use in bone regeneration as antibacterial membrane. The membrane was shown to have good antibacterial efficacy against both Gram-negative and Gram-positive bacteria, suggesting that it could be used in the prevention of infection in bone regeneration applications [143]. Over the years, systems for pulmonary drug delivery systems have been developed to treat lung disease. Jermi et al. designed a clay-based system with release capability of dexamethasone (Dex), to be used in coronavirus disease (COVID-19) treatment. They designed the system ZnFe_2_O_4_/Hal/DEX/PEG (Dex 5% wt/wt) sensitive to pH 5.6, able to release Dex at pulmonary infectious pH conditions [144]. Bordini et al. have developed an injectable GelMA-based nanotube modified hydrogel for controlled release of dexamethasone, illustrating the potential of this system for mineralized tissue regeneration. The DEX-loaded nanotube modified GelMA hydrogel (GelMA + 5.0%HNT-DEX10%) showed relevant properties from a mechanical point of view, but also biodegradability and cytocompatibility with mesenchymal stem cells from human exfoliated deciduous teeth (SHEDs). Moreover, the system had in vivo biocompatibility, and it also supported bone regeneration in vivo [145]. Phototherapy is described as a safe and secure way to destroy cancer cells when light waves of a particular wavelength are used with appropriate activating agents. Li et al. developed halloysite nanotubes decorated with poly(sodium-p-styrenesulfonate) (PSS) to enhance the biocompatibility, and further functionalized by lumen loading the type-II photosensitizer indocyanine green (ICG) to obtain a biomimetic nanocarrier platform for target-specific delivery of phototherapeutic agents. The obtained system, HNT-PSS-ICG, showed an excellent in vivo phototherapeutic effect against breast cancer in model mice [146]. Tan et al. developed a system of HNT-based multifunctional nanoparticles designed for tumour targeting and phototherapy in breast cancer treatment. Fluorescein isothiocyanate (FITC) was adsorbed on the HNT surfaces, and indocyanine green (ICG) was loaded as the photothermal agent into the lumen. To enhance the biocompatibility, the system was wrapped with red blood cell membrane (RBCM). Finally, anti-EpCAM was conjugated with HNTs-FITC-ICG-RBCM with the assistance of streptavidin to improve the specific uptake of breast cancer cells. The cell viability assay results for MCF-7 cells indicated that the HNTs-FITC-ICG-RBCM cytotoxicity was irradiation time and concentration dependent and that this could be potentially used in breast cancer treatment [147]. As evidenced by Lisuzzo et al., HNTs are excellent candidates as interfacially active inorganic particles for the formation of Pickering emulsions thanks to their advantages, as for example low cost, biocompatibility, mechanical strength, tubular morphology, etc. [148]. The functionalization of HNT surfaces allows the synthesis of infinite nano-architectures which have different properties and can be modulated according to the desired performance and the field of application. It is, therefore, clear why it has led to a deepening of the research for their possible applications.

## 6. Conclusions

Halloysite nanotubes are naturally occurring and cost-effective nanomaterials that are finding applications in several areas, some discussed here. Several types of functionalisation of HNT have been discussed, including the advantageous tubular structure that has given it numerous roles as drug delivery and gene delivery agents or nanocarriers. Recent studies demonstrate the potential of halloysite clay nanotubes for life science applications since results suggest that HNTs are a safe nanomaterial which can be used in biomaterials without serious side effects. However, more studies are needed to clarify the in vivo outcomes of long and chronic oral exposure to HNTs, which seems to depend on the administered concentration. Overall, the results obtained to date open wide prospects of investigation to better understand the use of these systems for a potential application as drug carrier and delivery systems. The combination of the innumerable properties of halloysite nanotubes, together with their biocompatibility and the possibility of functionalising the surfaces, makes them ideal candidates for the development of an innovative therapeutic approach.

## Figures and Tables

**Table 1:** Cytotoxicity of HNTs towards different cell lines.

Cell Type	Incubation Time	HNT Concentration	Reference
MCF7, HeLa	24–72 h	No toxic effect up to 75 μg mL^−1^	[25]
A549	24 h	No toxic effect up to 100 μg mL^−1^	[82]
Caco-2, HT29-MTX	6 h	No toxic effect up to 100 μg mL^−1^	[83]
A549	24–48–72 h	No toxic effect up to 100 μg mL^−1^	[84]
C6	24 h	No toxic effect up to 500 μg mL^−1^	[85]
Colo 320	24 h	No toxic effect up to 625 μg mL^−1^	[86]

**Table 2:** HNT nanocomposites for controlled and sustained anticancer drug delivery.

Anticancer Drug	Cell Type	HNT Modifications	Reference
Anthocyanins	MCF-7, HT-29	HNT-Anth	[114]
Atorvastatin	Caco-2, HT-29	HNT-ATV@HF-CEL	[115]
Camptothecin	HeLa	f-HNT/CPT and Fmoc-F/f-HNT/CPT	[116]
Camptothecin	Caco-2	CPT@COS/MHNTs and CPT@FA-COS/MHNTs	[117]
Curcumin	Caco-2	HNT-APT-PMVEMA@MF	[118]
Curcumin	HepG2, MCF-7, SV-HUC-1, EJ, CaSki, HeLa	HNT-COOH/Chitosan	[119]
Curcumin	SUM 149, MDA-MB-231, HL60, HL60R	f-Hal-1, 2, 4, 5, 6, and 7	[120]
Curcumin	MCF-7	PCL/PEO-Cur/HNT, PCL/PEO-Cur/HNT-GPTMS, and PCL/PEO-Cur/HNT-APTES	[121]
Doxorubicin	MCF-7	DOX@HNTs-g-COS	[122]
Doxorubicin	A549	DNA-wrapped HNTs	[123]
Doxorubicin	SKOV3, 293T	DOX@HNTs-S-S-β-CD-Ad-PEG-FA	[124]
Doxorubicin	MCF-7	DOX@HNTs-PEG-FA	[125]
Doxorubicin	HeLa	DOX loaded Fe_3_O_4_@HNT	[126]
Doxorubicin	MCF-7	Au-HNT-DOX@BSA-FA	[127]
Doxorubicin	MCF-7, COLO 205	HNT-liposome-coated surfaces	[128]
Doxorubicin	HeLa, MCF-7	HNTs-DOX conjugated with anti-EpCAM antibody	[129]

## Data Availability

Not applicable.
